# TB morbidity estimates overlook the contribution of post-TB disability: evidence from urban Malawi

**DOI:** 10.1136/bmjgh-2021-007643

**Published:** 2022-05-19

**Authors:** Ewan M Tomeny, Rebecca Nightingale, Beatrice Chinoko, Georgios F Nikolaidis, Jason J Madan, Eve Worrall, Lucky Gift Ngwira, Ndaziona Peter Banda, Knut Lönnroth, Denise Evans, Jeremiah Chakaya, Jamie Rylance, Kevin Mortimer, S. Bertel Squire, Jamilah Meghji

**Affiliations:** 1 Department of Clinical Sciences, Liverpool School of Tropical Medicine, Liverpool, UK; 2 Malawi-Liverpool-Wellcome Clinical Research Programme, Blantyre, Malawi; 3 EMEA Real World Methods and Evidence Generation, IQVIA, Durham, UK; 4 Warwick Clinical Trials Unit, Warwick Medical School, University of Warwick, Coventry, UK; 5 Department of Vector Biology, Liverpool School of Tropical Medicine, Liverpool, UK; 6 University of Malawi College of Medicine, Blantyre, Malawi; 7 Queen Elizabeth Central Hospital, Blantyre, Malawi; 8 Department of Global Public Health, Karolinska Institute, Stockholm, Sweden; 9 Health Economics and Epidemiology Research Office, Department of Internal Medicine, School of Clinical Medicine, Faculty of Health Sciences, University of the Witwatersrand, Johannesburg, South Africa; 10 Department of Medicine, Therapeutics, Dermatology and Psychiatry, Kenyatta University, Nairobi, Kenya; 11 International Union Against Tuberculosis and Lung Disease, Paris, France; 12 Department of Respiratory Medicine, Liverpool University Hospitals NHS Foundation Trust, Liverpool, UK

**Keywords:** tuberculosis, health economics, health policy, cohort study, indices of health and disease and standardisation of rates

## Abstract

**Introduction:**

Despite growing evidence of the long-term impact of tuberculosis (TB) on quality of life, Global Burden of Disease (GBD) estimates of TB-related disability-adjusted life years (DALYs) do not include post-TB morbidity, and evaluations of TB interventions typically assume treated patients return to pre-TB health. Using primary data, we estimate years of life lost due to disability (YLDs), years of life lost due to premature mortality (YLL) and DALYs associated with post-TB cardiorespiratory morbidity in a low-income country.

**Methods:**

Adults aged ≥15 years who had successfully completed treatment for drug-sensitive pulmonary TB in Blantyre, Malawi (February 2016–April 2017) were followed-up for 3 years with 6-monthly and 12-monthly study visits. In this secondary analysis, St George’s Respiratory Questionnaire data were used to match patients to GBD cardiorespiratory health states and corresponding disability weights (DWs) at each visit. YLDs were calculated for the study period and estimated for remaining lifespan using Malawian life table life expectancies. YLL were estimated using study mortality data and aspirational life expectancies, and post-TB DALYs derived. Data were disaggregated by HIV status and gender.

**Results:**

At treatment completion, 222/403 (55.1%) participants met criteria for a cardiorespiratory DW, decreasing to 15.6% after 3 years, at which point two-thirds of the disability burden was experienced by women. Over 90% of projected lifetime-YLD were concentrated within the most severely affected 20% of survivors. Mean DWs in the 3 years post-treatment were 0.041 (HIV-) and 0.025 (HIV+), and beyond 3 years estimated as 0.025 (HIV-) and 0.010 (HIV+), compared with GBD DWs of 0.408 (HIV+) and 0.333 (HIV-) during active disease. Our results imply that the majority of TB-related morbidity occurs *post-treatment*.

**Conclusion:**

TB-related DALYs are greatly underestimated by overlooking post-TB disability. The total disability burden of TB is likely undervalued by both GBD estimates and economic evaluations of interventions, particularly those aimed at early diagnosis and prevention.

**What is already known on this topic**

- It is increasingly clear that tuberculosis (TB) disease is associated with long-term physical and psychosocial sequelae, affecting patient health-related quality of life (HRQoL) long after treatment completion and microbiological cure.
- While Global Burden of Disease estimates were updated in 2019 to include a longer period of TB illness duration and excess mortality prior to treatment completion, they remain focused on morbidity during TB illness and treatment only and do not include morbidity in the post-TB period.
- Using a hypothetical cohort, a 2021 modelling study estimated that approximately half the global TB burden occurs post-treatment; however, morbidity data from longitudinal post-TB cohorts are needed to provide empirical evidence to support post-TB disability estimates.

**What this study adds**

- Using HRQoL data from a prospective cohort study, we have quantified TB-related disability occurring beyond treatment completion, demonstrating that—even with conservative estimates—the inclusion of the post-TB period would dramatically increase TB morbidity estimates.

**How this study might affect research, practice and/or policy**

- To accurately capture TB’s impact, researchers must ensure the inclusion of TB-related morbidity occurring in the post-TB period, a vital adjustment for ensuring appropriate allocation of resources to and within TB services, emphasising interventions which support disease prevention and early diagnosis.
- Mitigating post-TB morbidity should be highlighted as a priority for TB programmes, requiring programmatic guidelines for the clinical management of sequelae, alongside investment in post-TB data collection, reporting mechanisms, rehabilitation and social and economic support.

## Introduction

Tuberculosis (TB) remains an important cause of morbidity and mortality worldwide with an estimated 10 million incident cases of TB disease in 2020, causing 1.5 million deaths.^1^ Those who survive TB disease face a considerable but under-recognised burden of ongoing morbidity including respiratory impairment,^2–5^ psychosocial challenges and reduced health-related quality of life (HRQoL) after treatment completion.^6–14^ Guidelines for TB management define ‘treatment success’ using microbiological outcomes and survival only, and measures of TB-associated morbidity remain solely focused on the period prior to and during treatment.^15^

The most widely used measure of disease burden-associated morbidity is the disability-adjusted life year (DALY).^16^ This combines ‘years of life lost due to premature mortality’ (YLL) from a disease, with ‘years of life lost due to disability’ (YLD), with the latter calculated by multiplying the number of years lived in a certain health state by a ‘disability weight’ (DW) attributed to this state.^17^ The WHO recommends the use of DALYs in generalised cost-effectiveness analysis, prioritising interventions which offer a favourable ratio of DALYs averted to cost.^18^

Although there were an estimated 155 million TB-survivors alive in 2020,^19^ measures of YLD due to TB disease do not include post-TB sequelae, and consider disability during TB disease and treatment only. The Global Burden of Disease (GBD) study’s estimates assume that health returns to normal at treatment completion, with the time taken to reach this point weighted by each country’s Health Access and Quality Index.^20^ The DWs allocated to active TB disease, with and without HIV coinfection, in the 2019 GBD study are 0.408 (0.274–0.549) and 0.333 (0.224–0.454), respectively (online supplemental appendix table A1).^21^ These DWs lead to an annual estimate of YLD for drug-sensitive TB disease of 4.8 million years globally.

10.1136/bmjgh-2021-007643.supp1 Supplementary data

Given the growing body of evidence for post-TB sequelae, we hypothesised that by not accounting for residual disability after treatment completion, the GBD study and TB-related economic evaluations underestimate both the YLD from each case of TB disease, and the value of interventions to prevent TB disease or optimise the care-cascade.

Post-TB lung damage is a key form of post-TB morbidity, seen in over a third of TB survivors, and associated with chronic respiratory symptoms, functional impairment and health seeking.^2–12^ Recent work aiming to calculate the YLD for HIV-negative TB survivors has estimated DWs associated with post-TB morbidity based on cross-sectional prevalence estimates of Chronic obstructive pulmonary disease (COPD) and chronic respiratory diseases among recovered patients with TB.^22^ While one further 2010 American study has also suggested post-TB DWs,^23^ to date, there have been no studies which directly derive post-TB DWs from longitudinal HRQoL data from patients in a high-TB incidence, low-income setting. The aim of this study is to address this evidence gap.

In this study, we use data from a prospective cohort of adult pulmonary tuberculosis (PTB) survivors in Malawi to directly quantify the burden of cardiorespiratory YLD associated with each case of PTB, in the period after TB treatment completion. We derive post-TB cardiorespiratory DWs, YLDs and DALYs for HIV-positive and HIV-negative members of this population and highlight the considerable global implications for post-TB care.

## Methods

### Study population

This study is a secondary analysis of post-TB respiratory morbidity data from a prospective cohort study of adults completing treatment for drug-sensitive PTB in urban Blantyre, Malawi. The full study design and respiratory findings are published elsewhere.^4 24^ In brief, participants aged ≥15 were prospectively recruited at TB treatment completion (T_0_) (February 2016–April 2017). Demographic data and HIV status were recorded at T_0_, and questionnaire data and respiratory measurements collected at 6-month and 12-month intervals (T_0_, T_6_, T_12_, T_24_, T_30_, T_36_) over 3 years. HRQoL was assessed at each time point using a Chichewa translation of the St George’s Respiratory Questionnaire (SGRQ), a respiratory-focused specific instrument validated for use in post-TB populations, which provides a score from 0 to 100.^25^ A validated Chichewa version of the EQ-5D-3L*—a generic HRQoL tool*—was also administered at T_0_, T_6_ and T_12_.^26^

### Study design

#### Examining HRQoL changes

Data were disaggregated by HIV status and gender. The proportion of participants who experienced deterioration, improvement or ‘no change’ in health between consecutive study visits was determined using a minimal clinically important difference of 4 points in SGRQ ‘total score’ (hereafter *SGRQ score*).^25^ Participants were divided into severity quintiles based on SGRQ score at T_0_, to observe trends in HRQoL over time.

#### Calculating DWs: 3-year period

GBD methodology assigns DWs to health states in a process that uses ‘lay-descriptions’ which ‘*emphasise the major functional consequences and symptoms associated with each health state*’ rather than specifying underlying diseases or diagnoses,^17^ such that DWs can reasonably be applied across disease entities based on the presence of symptoms and impairment alone. All 235 health states described within the GBD study were reviewed (online supplemental appendix A).^17^ Twelve cardiorespiratory states were identified for which corresponding SGRQ symptom data were available from our cohort, with DWs ranging from 0.015 to 0.408. Health state mapping was performed for each participant at each time point (T_0_→T_36_), by relating the symptoms detailed in these lay descriptions, to those collected by the SGRQ and allocating the corresponding DW (DW_0_→DW_36_). A conservative approach was taken, whereby only patients reporting *all symptoms* described for each health state were allocated the corresponding DW (online supplemental appendix B), with sensitivity analyses conducted using less stringent criteria. Where participants satisfied criteria for two or more DWs at a given time point, the ‘*maximum limit method’* was followed,^27^ assigning the largest DW among satisfied states, to give their ‘maximum DW’.

Mean DWs were calculated for each severity quintile at each time point and trends in progression compared with those seen in SGRQ, Visual Analogue Scale (EQ-VAS) and EQ-5D-3L index values.

#### Calculating YLD: 3-year period

YLD were directly derived from study data for the 3 years of cohort follow-up. DWs at discrete study visits were translated to a continuous measure of 3-year YLD using an approach equivalent to calculating the ‘area under the curve’^28^: transitions between DWs at consecutive time points were assumed to happen at a constant rate, and YLD in each interval calculated by multiplying the intermediary period length (typically 0.5 years) by the mean of the DWs at adjacent visits. In a small number of cases where HRQoL data were not available for a single time point, DWs were averaged from the nearest available time points. The 3-year YLD for each participant was calculated as the sum of YLD across all intervals (online supplemental appendix figure C1). Variances in disability progression by HIV status and gender were explored, with differences evaluated using t tests. Primary YLD analyses were completed using a ‘*core sample*’ of those who completed the full 3-year follow-up and for whom baseline data were available (n=299), with sensitivity analyses conducted using aggregated population means of all participants.

#### Estimating YLD: lifetime

Lifetime YLD were projected for the period after the 3-year study follow-up, for the *core sample* only. To explore the trajectory in HRQoL, various models were fit to mean SGRQ scores from T_0_ to T_36_ (online supplemental appendix D). A quadratic plateau model provided the best fit, which—along with decreasing clinically significant SGRQ changes between subsequent time points—supported stability of morbidity by T_36_. Accordingly, disability at this time point (DW_36_) was assumed to remain constant after the 3-year study period. Each participant’s ongoing YLD was calculated by multiplying DW_36_ by their estimated life years remaining, with the latter estimated by subtracting age at T_36_ from their life expectancy given in the Malawi life table (by sex and age).^29^ No adjustment was made for elevated mortality ratios of TB-survivors.^30^

Total post-TB YLD for each participant was calculated by adding the data-derived 3-year YLD to the lifetime projection of YLD.

#### Years of life lost due to premature mortalities

While our study’s focus is on post-TB disability, for completeness, we estimated YLLs for our population of patients with post-TB. Cause of death was not available in our data, and we ran our analyses two times using two sets of assumptions. First, we assumed all deaths identified during the 3-year study period were TB related, and second, we assumed only the deaths of those known to be undergoing TB retreatment had been due to TB. In each case, YLL were calculated using aspirational life expectancy at the age of death, taken from the 2019 GBD reference life table.^31^ We took a conservative approach, assuming no additional TB-related deaths among those not completing follow-up and no incremental mortality risk after the follow-up period.

#### Total DALYs

YLD during the 3-year study period were inferred for those not completing follow-up. These participants were assigned the mean disability for the 3-year period of those within the ‘core sample’ with the same HIV status and of the same gender. Three-year YLD totals include the predeath disability experienced by those who died. Total DALYs were estimated separately for HIV-positive and HIV-negative participants by summing the estimated study period YLD, the estimated remainder of life YLD and YLL (presented with our first set of assumptions).

#### Comparison of active disease and post-TB disability

To understand the wider implications of our findings, we compared our estimates of post-TB YLD to those which might have been calculated for these patients during active disease only, using the GBD DWs of 0.408 HIV-positive and 0.333 HIV-negative provided for active drug-susceptible disease (online supplemental appendix table A1).^21^ We used mean durations of 1.1 years (HIV-negative) and 0.51 years (HIV-positive), taken from the duration assumptions used in the WHO’s TB burden estimations,^32^ and in line with those typically applied in TB cost-utility studies (online supplemental appendix table E1).

Analyses were conducted in Stata V.14.0 (Stata Corp.2015, College Station, Texas) and RStudio V.1.2.5 (Boston, Massachusetts).

#### Patient and public involvement

Data from the original cohort study have been shared with participants, patient advocacy groups, healthcare providers and the National TB programme in Malawi. These groups are involved in an ongoing programme of stakeholder engagement around strategies for post-TB care in Eastern Africa, which is being led by the study team (NIHR130307).

## Results

### Study population

405 participants (275 men and 130 women) were recruited, with 299 completing full 3-year follow-up (the *‘core sample’*), including 292 participants interviewed at all six time points (T_0_→T_36_)(online supplemental appendix figure F1). At baseline, 60.5% (244/403) were HIV positive (2/405 unknown), and 77.3% (313/405) had had microbiologically proven PTB. Mean age was 32.4 years for women (SD: 10.2), and 36.2 years for men (SD: 10.0). Cohort demographics and detailed respiratory parameters are published elsewhere.^4 24^

### HRQoL progression

**Figure 1:**
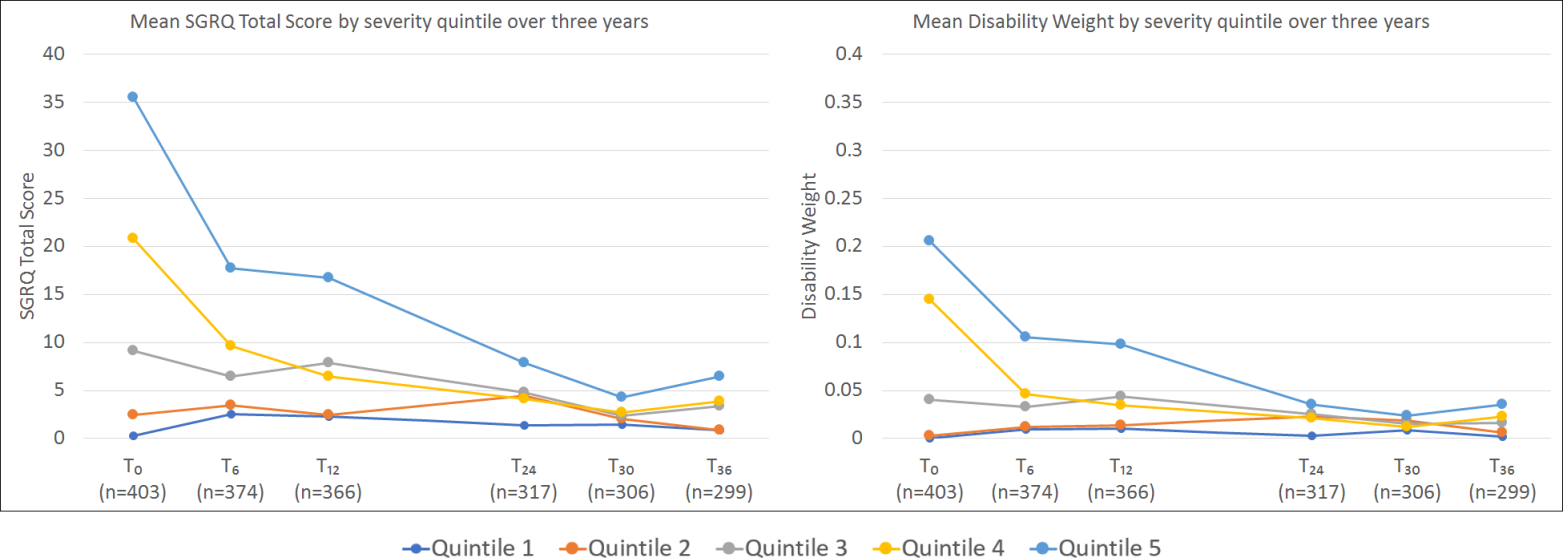
Changes in mean SGRQ score and mean disability weight over 3-year study period by SGRQ severity quintile at TB treatment completion.SGRQ scores (total scores) can range from 0 to 100. The displayed severity quintiles (quintile 1 least severe; quintile 5 most severe) were formed by ranking all participants by their SGRQ score at baseline. Included in quintile 1 are 27 participants who scored 0 at baseline (T_0_). The most marked improvement was seen among those with highest baseline scores, however, on average, these participants still had the worst scores at T_36_. For similar graphs showing EQ-5D and VAS for T_0_ - T_12_, see online supplemental appendix figure F3. SGRQ, St George’s Respiratory Questionnaire; VAS, Visual Analogue Scale.

SGRQ scores at T_0_ ranged from 0 to 56.7 with median 8.8 (IQR 1.3–23.4). Data were moderately right-skewed, with 22.8% scoring 0 at T_0_. This proportion increased to 51.2% by T_36_, with data highly right-skewed (skewness 3.54). There were no statistically significant differences in EQ-VAS (p=0.188), EQ-5D (p=0.208) and SGRQ (p=0.064) scores by HIV status at T_0_. Across all HIV status/gender subgroups, the greatest improvement in health was seen in the 6-month post-treatment (online supplemental appendix figure F2 and table F1). When SGRQ progression was examined by baseline severity, improvements were seen across all severity quintiles (figure 1).

### DWs: 3-year period

A total of 222 participants (55.1%) satisfied the criteria for at least one of the 12 identified cardiorespiratory DWs at TB treatment completion, with State*_HF_MILD_* and State*_COPD_MILD_* satisfied by 52.6% and 52.4% of participants, respectively (online supplemental appendix figure F2). The most common (maximum) disability states at T_0_ were State*_COPD_MOD_* (23.3%) and State_HF_MOD_(10.7%) (table 1).

**Table 1:** Allocated health states and maximal disability weights at each time point

Health state name^17^ (Health state ID)	Health state lay description	Number for whom this disease state is their worst state at each time point (n, %)						
		GBD disability weight (uncertainty interval)	T_0_ (n=403)	T_6_ (n=376)	T_12_ (n=368)	T_24_ (n=319)	T_30_ (n=308)	T_36_ (n=301)
Acute myocardial infarction, days 3–28 (State_ACUTE_MI_)	Gets short of breath after heavy physical activity, and tires easily, but has no problems when at rest. The person has to take medication every day and has some anxiety.	0.074 (0.049–0.105)	1 (0.2%)		1 (0.3%)	1 (0.3%)		
Anaemia, moderate (State_ANEMIA_MOD_)	Feels moderate fatigue, weakness, and shortness of breath after exercise, making daily activities more difficult.	0.052 (0.034–0.076)						2 (0.7%)
Anaemia, severe (State_ANEMIA_SEV_)	Feels very weak, tired and short of breath, and has problems with activities that require physical effort or deep concentration.	0.149 (0.101–0.209)	37 (9.2%)	4 (1.1%)	11 (3.0%)	2 (0.6%)	1 (0.3%)	2 (0.7%)
Asthma, controlled (State_ASTHMA_CON_)	Has wheezing and cough once a month, which does not cause difficulty with daily activities.	0.015 (0.007–0.026)	1 (0.2%)	4 (1.1%)	1 (0.3%)	4 (1.3%)	3 (1.0%)	1 (0.3%)
Asthma, partially controlled (State_ASTHMA_PAR_CON_)	Has wheezing and cough once a week, which causes some difficulty with daily activities.	0.036 (0.022–0.055)						
Asthma, uncontrolled (State_ASTHMA_UNCON_)	Has wheezing, cough and shortness of breath more than twice a week, which causes difficulty with daily activities and sometimes wakes the person at night.	0.133 (0.086–0.192)						
COPD and other chronic respiratory problems, mild (State_COPD_MILD_)	Has cough and shortness of breath after heavy physical activity, but is able to walk long distances and climb stairs.	0.019 [0.011–0.033	4 (1.0%)		1 (0.3%)	2 (0.6%)		1 (0.3%)
COPD and other chronic respiratory problems, moderate (State_COPD_MOD_)	Has cough, wheezing and shortness of breath, even after light physical activity. The person feels tired and can walk only short distances or climb only a few stairs.	0.225 (0.153–0.310)	94 (23.3%)	52 (13.8%)	47 (12.8%)	20 (6.3%)	14 (4.5%)	14 (4.7%)
COPD and other chronic respiratory problems, severe (State_COPD_SEV_)	Has cough, wheezing and shortness of breath all the time. The person has great difficulty walking even short distances or climbing any stairs, feels tired when at rest, and is anxious.	0.408 (0.273–0.556)				1 (0.3%)		
Heart failure, mild (State_HF_MILD_)	Is short of breath and easily tires with moderate physical activity, such as walking uphill or more than a quarter-mile on level ground. The person feels comfortable at rest or during activities requiring less effort.	0.041 (0.026–0.062)	42 (10.4%)	40 (10.6%)	29 (7.9%)	23 (7.2%)	21 (6.8%)	21 (7.0%)
Heart failure, moderate (State_HF_MOD_)	Is short of breath and easily tires with minimal physical activity, such as walking only a short distance. The person feels comfortable at rest but avoids moderate activity.	0.072 (0.047–0.103)	43 (10.7%)	16 (4.3%)	9 (2.4%)	4 (1.3%)	6 (1.9%)	6 (2.0%)
Heart failure, severe (State_HF_SEV_)	Is short of breath and feels tired when at rest. The person avoids any physical activity, for fear of worsening the breathing problems.	0.179 (0.122–0.251)						
Any disability weight	–	–	222 (55.1%)	116 (30.9%)	99 (26.9%)	57 (17.9%)	45 (14.6%)	47 (15.6%)

COPD, Chronic obstructive pulmonary disease; GBD, Global Burden of Disease.

**Figure 2:**
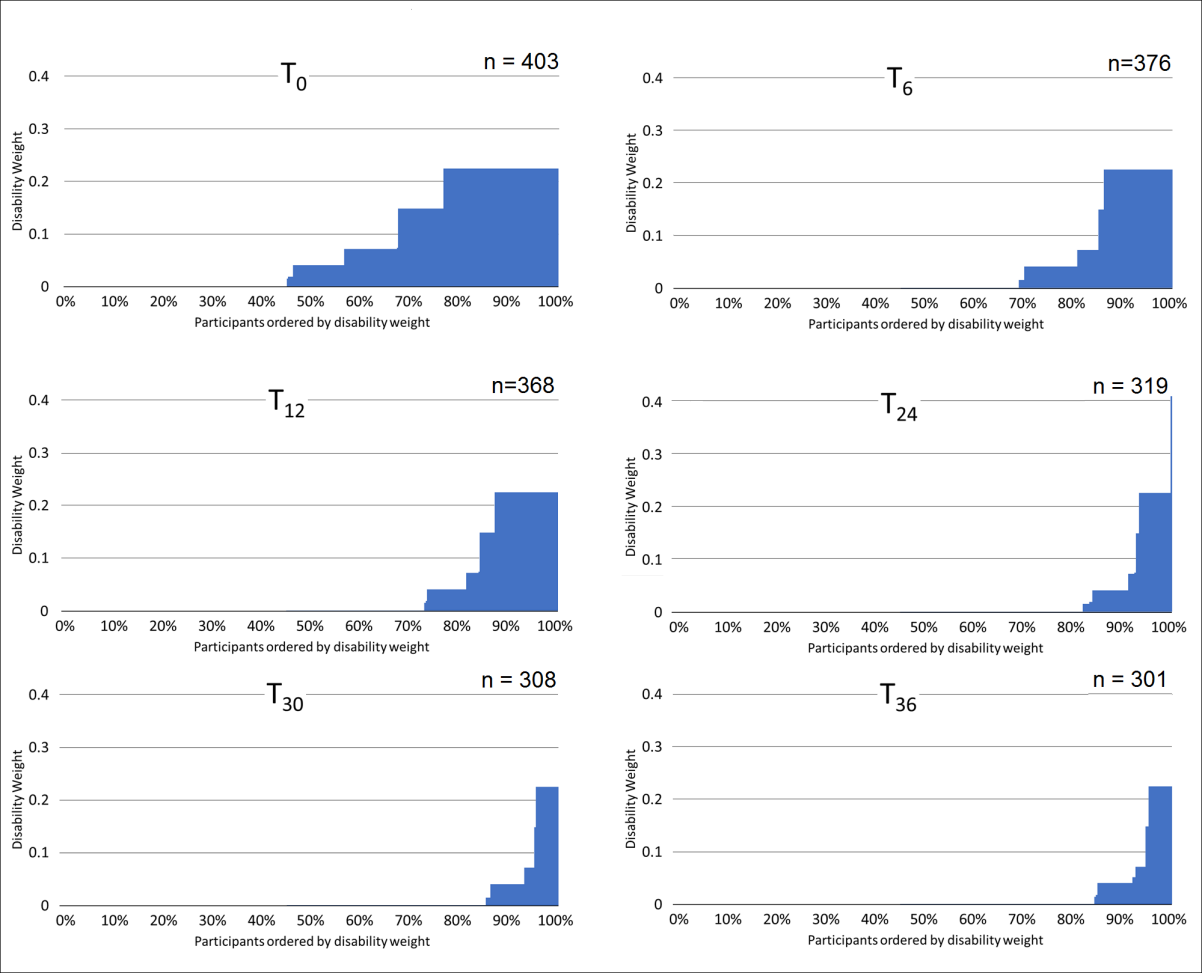
Maximum disability weights attributed to each participant, at each time point. Participants ordered from smallest to largest disability weight, with the assigned disability weight of each represented by a vertical blue bar. For participants who satisfied criteria for two or more health conditions at a single time point, they were assigned the largest disability weight among these (ie, the ‘maximum disability weight’).

DWs demonstrated clear discrimination between SGRQ severity quintiles at each time point, with mean weights tracking SGRQ scores (figure 1). Consistent with SGRQ score, the proportion of participants fulfilling criteria for states fell with each visit until T_30_. The largest decrease was seen immediately post-treatment (44% decrease T_0_→T_6_), and by T_30_ a nadir had been reached with 14.6% (45/308) of participants meeting a DW description (figure 2).

The mean DWs for men and women at T_0_ were 0.0771 (SD: 0.0934) and 0.0815 (SD: 0.0889), and at T_36_ were 0.0119 (SD: 0.0426) and 0.0248 (SD: 0.0611). However, at all time points, disability was concentrated in a minority of individuals (figure 2).

### YLD: 3-year period

The 3-year YLD across the core sample was 28.171, with mean 0.094 YLD (SD: 0.131) per participant, equivalent to 0.031 YLD per-year (table 2). Sensitivity analysis using the full data set and averages across time points calculated a similar 3-year mean disability of 0.096 YLD (+2%) per participant (online supplemental appendix table F4 and figure F4). At treatment completion, YLD were split proportionately by gender; however, by T_30_→T_36_ two-thirds of YLD were among women (table 3). The mean YLD of HIV-positive participants was lower than HIV-negative participants at baseline, with the relative difference growing over time. The 3-year DWs calculated for HIV-negative and positive participants were 0.041 (SD: 0.054) and 0.025 (SD: 0.033), respectively (table 2).

**Table 2:** Three-year study period and lifetime projections of YLD within *core sample*, disaggregated by the severity of HRQoL impairment at T_0_, and presented for the total cohort, and stratified by gender and HIV status

		Disability over the 3- year follow-up period				Projected lifetime disability (including 3-year post-treatment)				Disability weights	
		N	Mean YLD	Total YLD	Burden	N	Mean YLD	Total YLD	Burden	During 3 years	After 3 years
All participants in *core sample* (n=299)	*Least severe 20%	94	0.000	0.000	0%	94	0.000	0.000	0%	0.000	0.000
	–	30	0.012	0.371	1%	29	0.012	0.360	0%	0.004	0.000
	–	55	0.041	2.238	8%	57	0.042	2.419	1%	0.014	0.000
	–	60	0.111	6.672	24%	60	0.154	9.253	5%	0.037	0.000
	Most severe 20%	60	0.315	18.890	67%	59	3.062	180.629	94%	0.105	0.083
	**All 3-year participants**	**299**	**0.094**	**28.171**	**100%**	**299**	**0.644**	**192.662**	**100%**	**0.031**	**0.016**
Men (n=198)	Least severe 20%	70	0.000	0.000	0%	38	0.000	0.000	0%	0.000	0.000
	–	11	0.009	0.102	1%	10	0.009	0.092	0%	0.003	0.000
	–	38	0.038	1.427	8%	38	0.038	1.438	2%	0.013	0.000
	–	41	0.107	4.390	25%	40	0.134	5.365	6%	0.036	0.000
	Most severe 20%	38	0.307	11.661	66%	40	2.096	83.845	92%	0.102	0.060
	**All men**	**198**	**0.089**	**17.580**	**100%**	**198**	**0.458**	**90.739**	**100%**	**0.030**	**0.012**
Women (n=101)	Least severe 20%	24	0.000	0.000	0%	24	0.000	0.000	0%	0.000	0.000
	–	19	0.015	0.286	3%	19	0.015	0.286	0%	0.005	0.000
	–	18	0.048	0.867	8%	18	0.050	0.901	1%	0.016	0.000
	–	21	0.139	2.912	27%	20	0.224	4.483	4%	0.046	0.001
	Most severe 20%	19	0.343	6.525	62%	20	4.813	96.253	94%	0.114	0.125
	**All women**	**101**	**0.105**	**10.591**	**100%**	**101**	**1.009**	**101.923**	**100%**	**0.035**	**0.025**
HIV-positive participants (n=179)	Least severe 20%	57	0.000	0.000	0%	57	0.000	0.000	0%	0.000	0.000
	–	19	0.013	0.246	2%	19	0.013	0.246	0%	0.004	0.000
	–	35	0.039	1.373	10%	33	0.038	1.268	2%	0.013	0.000
	–	32	0.090	2.876	22%	34	0.108	3.663	5%	0.030	0.000
	Most severe 20%	36	0.243	8.759	66%	36	2.069	74.471	93%	0.081	0.052
	**All HIV positive**	**179**	**0.074**	**13.254**	**100%**	**179**	**0.445**	**79.649**	**100%**	**0.025**	**0.010**
HIV-negative participants (n=119)	Least severe 20%	37	0.000	0.000	0%	37	0.000	0.000	0%	3.333	0.000
	–	11	0.011	0.125	1%	10	0.011	0.115	0%	8.000	0.000
	–	23	0.048	1.113	8%	24	0.055	1.317	1%	8.000	0.000
	–	24	0.161	3.852	26%	24	0.269	6.451	6%	8.000	0.002
	Most severe 20%	24	0.400	9.593	65%	24	4.321	103.714	93%	39.667	0.122
	**All HIV negative**	**119**	**0.123**	**14.683**	**100%**	**119**	**0.938**	**111.597**	**100%**	**0.041**	**0.025**

Shaded cells show average DWs for each subgroup, appropriate for YLD calculations in an economic evaluation. They are calculated as the mean 3-yearYLD divided by three. For HIV status within gender, see online supplemental appendix table F3.

*Note least severe groupings larger due to number of participants measuring no impairment at T0. Further note, grouping by HIV status does not include one participant who declined testing.

HRQoL, health-related quality of life; YLD, years of life lost due to disability.

**Table 3:** YLD across time points split first by gender and second by HIV status

	Male		Female		Ratio of male to female mean disability	p-value for difference in disability between groups
	Number of participants	Mean YLD	Number of participants	Mean YLD		
T_0_–T_6_	255	0.029	123	0.030	49:51	0.803
T_6_–T_12_	249	0.018	119	0.021	47:53	0.542
T_12_–T_24_	212	0.026	107	0.033	44:56	0.301
T_24_–T_30_	208	0.007	105	0.013	36:64	0.028*
T_30_–T_36_	200	0.006	101	0.012	34:66	0.013*
**T**_**0**_ **–T**_**36**_	**101**	**0.105**	**198**	**0.089**	**46:54**	**0.316**

	HIV positive		HIV negative		Ratio of HIV positive to HIV negative disability	p-value* for difference in disability between groups
	Number of participants	Mean YLD	Number of participants	Mean YLD		
T_0_–T_6_	231	0.026	145	0.034	43:57	0.022*
T_6_–T_12_	225	0.017	142	0.023	42:58	0.063
T_12_-T_24_	192	0.023	126	0.036	39:61	0.031*
T_24_–T_30_	187	0.006	125	0.012	34:66	0.016*
T_30_–T_36_	180	0.005	120	0.012	30:70	0.003*
**T**_**0**_ **–T**_**36**_	**179**	**0.074**	**119**	**0.123**	**38:62**	0.001*

*Significant with α=0.05.

YLD, years of life lost due to disability.

### YLD: lifetime

By T_36_, 47/299 (15.7%) of the core sample satisfied a DW description with mean 0.104. Among core participants, mean life years left from T_36_, as given by the Malawi life table, were 31.0 years (men) and 38.9 years (women). The estimated post-3-year YLD for the core sample was 164.5 YLD.

There were 192.7 YLD over the full post-treatment period, giving a mean of 0.644 (SD: 1.87) lifetime YLD per participant, equivalent to 0.02 (SD: 0.053) YLD per life year remaining. Sensitivity analyses demonstrated that relaxing our stringent requirements for mapping states would have resulted in many more participants assigned disease states, and subsequently considerably larger disability estimates (online supplemental appendix table F5).

At a population level, 85.4% (164.5/192.7) of all YLD were incurred in the post-3-year period. Across all subgroups (men, women, HIV negative, HIV positive), 94% of full lifetime YLD were concentrated within the most severe 20% of participants.

### Years of life lost due to premature mortality

104/405 participants were not interviewed at T_36_ and 22 of these participants were known to have died (16 men and 6 women). Twenty (91%) of those who died were HIV positive, with five participants known to be undergoing TB retreatment at time of death (four HIV+; one HIV-). When assuming all 22 deaths were TB related, we calculated 1016.7 YLL for HIV-positive participants (n=244) and 108.5 YLL for the HIV-negative participants (n=159). When assuming only deaths of those undergoing retreatment were due to TB, we calculated 187.6 YLL and 54.3 YLL for HIV-positive and HIV-negative participants.

### Total DALYs

Eighty-two participants did not complete follow-up and were not known to have died, and baseline SGRQ was unavailable for two further participants; there was no difference in baseline SGRQ between these 84 participants and the core sample (p=0.300). Post-TB disability was, therefore, inferred for these participants from the core sample, allocating subgroup means according to HIV status and gender (online supplemental appendix table F3).

Using these estimates and the larger of our YLL estimates, post-TB DALYs across the full baseline population totalled 1372.8 DALYs (1016.7 YLL +108.5 YLL +247.6 YLD). This includes 1114.8 DALYs for HIV-positive participants, 256.1 DALYs for HIV-negative participants, and 1.9 DALYs for HIV status unknown (n=2). Dividing by the population sizes (159 HIV-negative, 244 HIV-positive) gives means of 4.56 (HIV+) and 1.61 (HIV-) DALYs per participant. This reduced to 489.4 DALYs under the more conservative estimate of YLLs (241.8 YLL +247.6 YLD).

### Comparison of active disease and post-TB

For our core sample of 119 HIV-negative and 179 HIV-positive participants, inclusion of YLD in the 3-year post-treatment period increased TB-disability estimates by 34% and 36%, respectively, compared with those estimated for active disease only, while lifetime projections gave an increase over active disease of 256% and 214% (online supplemental appendix E).

## Discussion

Most adults successfully treated for PTB in urban Malawi have disability meeting the descriptions of GBD cardiorespiratory health states at treatment completion. While recovery is observed over time, 16% continue to satisfy at least one GBD disability state 3 years later. Our findings suggest that health plateaus after this 3-year time point, and assuming this level of disability then persists over the life course, we estimate a mean of 0.94 YLD per HIV-negative patient and 0.45 YLD per HIV-positive patient in the post-TB period. These compare to 0.59 YLD (HIV-) and 0.78 (HIV+) during active disease (derived from GBD data), with the majority of post-TB disability concentrated in a minority of people. Our results suggest currently unaccounted for post-TB morbidity makes a marked contribution to the morbidity of TB disease, and may, on average, exceed that experienced during active disease.

The average burden of disability experienced across this post-TB population is high. The GBD allocates DWs of 0.408 and 0.333 to active TB disease among HIV-positive and HIV-negative adults.^17^ Our study calculated average DWs of 0.025 and 0.041 for the 3-year period following treatment and estimated weights of 0.010 (HIV-positive) and 0.025 (HIV-negative) to apply thereafter. Our finding that HRQoL remains reduced following successful TB treatment is consistent with previous studies^7–9^ and with the degree of underlying post-TB respiratory pathology identified in this cohort and elsewhere.^4 33^

The weights derived in this study were smaller than those modelled by Quaife *et al* (0.053 for the post-TB period),^22^ and those reported by Pasipanodya *et al* which ranged from 0.173 for ‘non-impaired’ to 0.377 for ‘severely impaired’ survivors.^23^ However, we note that these studies did not account for HIV status, and estimated DWs using cross-sectional prevalence estimates of COPD in a post-TB HIV-negative population^22^ or calculated DWs at a single point 20 weeks post-therapy in a high-income country, with the exact methods used to scale from SGRQ responses to DWs not reported.^23 34^ Our weight derivation approach was carefully allied to a range of GBD weights, modelled progression over 3 years of primary data; the consistency observed across SGRQ score, EQ-VAS score and DWs over time suggests validity in our approach. However, these previous studies suggest that our study likely provides a conservative estimate of post-TB morbidity, making our findings even more striking.

We estimated 247.6 post-TB YLDs across our baseline population (n=405; 61% HIV-positive). To contextualise this estimate, we compared this disability to what might have been assumed during active disease for the participants in our sample. Remarkably, our YLD estimates for post-TB disability increased the mean TB-related disability estimates by 256% (HIV-negative) and 214% (HIV-positive). These values are in line with those estimated by Menzies *et al*, who*—notably also taking conservative assumptions*—estimated a mean increase of 383% (95% CI 154 to 755) in TB-related YLD globally, once morbidity experienced in the post-TB period had been added to that experienced during active disease.^35^ While the degree of TB disability will vary between countries—particularly associated to their income^13^—our results point towards a vast amount of post-TB disability being overlooked among the 155 million TB survivors worldwide.^19^

Our data suggest that post-TB morbidity is borne by a minority, with 94% of YLD faced by 20% of participants. This finding is consistent with the skewed distribution of respiratory pathology observed within this sample and has implications for approaches to post-TB care.^4 24^ When communicating to TB-affected communities, there is a need not only to acknowledge post-TB morbidity but also to emphasise that most adults completing treatment *do* in fact recover. Our findings indicate that therapeutic interventions may be best targeted at the minority with severe or persistent disease, where they would likely be most cost-effective.

Our study demonstrated higher disability among HIV-negative, compared with HIV-positive adults. This was observed at treatment completion, with differences widening over time. This is surprising, given our understanding of the detrimental effect of HIV on well-being, and the low CD4 count observed among the HIV-positive patients with TB.^36^ This might reflect an attrition bias, as our primary analysis excluded those who dropped out or died during follow-up, of whom 91% were HIV positive. However, sensitivity analysis including all patients at all time points provided a mean DW only 2.1% larger (0.0314 *vs* 0.0321), suggesting this was not responsible (online supplemental appendix table F4). We have previously reported more severe residual respiratory pathology among HIV-negative PTB survivors in this cohort,^4^ and as our DWs mainly pertain to respiratory symptoms, this difference may reflect the extent of underlying post-TB lung disease between groups.^37 38^

Also consistent with previous studies was our finding that YLD showed increasing differences between genders over time, with women bearing disproportionately more disability and reporting lower improvements in HRQoL (online supplemental appendix figure F5). A study of a South-Indian population 1-year post-treatment had reported lower social and mental well-being scores among women.^9^ Gender disparities in the diagnosis and management of TB disease are receiving increased attention,^39^ and our findings suggest that this agenda should aim to include the experience of post-TB morbidity.

Mortality among the TB survivors in this cohort was higher than expected at a population level. WHO life tables indicate that over our study period, one would expect fewer than three deaths among this sample if taken randomly from the Malawian population (reflecting size; age/gender distribution).^31^ Our finding of raised mortality is consistent with previous studies, which have shown increased mortality among TB survivors, compared with TB naïve adults^40^ and confirms that YLL post-TB must be considered alongside YLD if the true burden of TB disease is to be described.

Our study has several limitations. We focused on the cardiorespiratory sequelae of TB disease and did not include broader morbidity from extrapulmonary disease, drug side effects and psychosocial challenges. Recent studies have highlighted the broad extent of TB disability, and additional work to quantify these further types of morbidity is needed.^33^ Our approach also assumed constant disability beyond 3 years post-treatment, however, TB survivors may experience respiratory exacerbations,^4 40 41^ cardiorespiratory decline^24^ and recurrent TB disease,^42 43^ such that disability in a minority might worsen over time. Our analyses did not consider reduced life expectancy among TB survivors^44 45^ and importantly focused on residual morbidity among adult survivors of drug-sensitive PTB disease only. These limitations mean that our estimates of post-TB DALYs are likely conservative. Further data are needed from children, and those with multidrug-resistant and extrapulmonary disease, in urban and rural settings elsewhere. In the absence of cause of death data, our YLL (and therefore DALY) estimates required several assumptions, and data on cause of death among TB survivors are urgently needed to narrow these estimates. Finally, without a control group, it cannot be guaranteed that a participant’s post-TB disability was exclusively due to their TB. Our assumption of a causal link, however, is strengthened by the clear recovery patterns observed across the cohort following treatment completion, which are in keeping with recovery after severe illness such as TB disease.

This study also has important strengths. To our knowledge, no previous study has combined both HRQoL data from a cohort of TB survivors and GBD disability states and weights to directly derive DWs, YLD and DALYs for the post-TB period. By using primary HRQoL and mortality data from a large prospective cohort study of post-TB morbidity in Africa with extended follow-up, our novel approach ensured that our derived DWs are directly comparable to those used in the construction of DALYs for active TB and other diseases. Furthermore, our sensitivity analyses included comparisons between SGRQ and DW trajectories over time, and comparisons of YLD calculations between our full and core sample, and showed good consistency. Together, these factors suggest validity in both our analytical approach and findings. Finally, this study describes long-term morbidity after TB treatment completion and highlights a neglected period in the TB-patient journey.

## Conclusion

By excluding the post-TB period, existing GBD calculations substantially underestimate the true burden of TB-related morbidity. This has implications for resource allocation to TB services, and for cost-utility analyses of interventions aimed at TB prevention, early diagnosis, reducing transmission or addressing post-TB sequelae. The methodological approach introduced here, using HRQoL data to allocate GBD-comparable DWs, could be applied to data from other studies of post-TB morbidity, in order to further our understanding of the impact of the sequelae of TB disease.

## Data Availability

Data are available upon reasonable request. The code used in our analyses is available upon request.
