# Supplementary material for: TB morbidity estimates overlook the contribution of post-TB disability: evidence from urban Malawi
Source: BMJ Glob Health. 2022 May 19;7(5):e007643. doi: 10.1136/bmjgh-2021-007643 (PMC9125716; doi:10.1136/bmjgh-2021-007643)
Supplement: Supplementary data [file bmjgh-2021-007643supp001.pdf]

Appendices

Title: TB morbidity estimates overlook the contribution of post-TB disability : evidence from urban Malawi

Authors: Ewan M Tomeny, Rebecca Nightingale, Beatrice Chinoko, Georgios F Nikolaidis, Jason Madan, Eve Worrall, Lucky-Gift Ngwira, Ndaziona PK Banda, Knut Lönnroth, Denise Evans, Jeremiah Chakaya Muhwa, Jamie Rylance, Kevin Mortimer, S. Bertel Squire, Jamilah Meghji

Contents

Appendix A: Selection of relevant GBD Disease States 2
Appendix B: Mapping study SGRQ data to GBD states 4
Appendix C: Proceeding from health states to calculating DALYs 8
Appendix D: Modelling lifetime disability 9
Appendix E: Comparison of active disease and post-TB disability 11
Appendix F: Additional Tables and Figures 12

Appendix A: Selection of relevant GBD Disease States

Since the first GBD report (1996), many studies have generated disability weights sets. Methods vary, but typically use 'disease-specific' descriptions of health states, a panel of 'judgers' (either medical experts, affected patients, or general population), and a non-preference based valuation method administered by either questionnaire, interview, or panel meeting. The 235 unique health states evaluated in the 2013 GBD '*Disability Weights Measurement Study'* were calculated by presenting members of the public with short and simple (non-medical) descriptions of health states, and then eliciting their relative preferences through pairwise comparisons. A relative ranking was then formed, and each unique state assigned a weight, anchored using an additional valuation question. Despite being considered 'disease specific', those valuing did not see names, only lay-descriptions which '*emphasised the major functional consequences and symptoms associated with each health state*', meaning one health state can be used to describe the experience of several diseases or conditions. Accordingly, a disability weight initially 'intended' to represent one condition, e.g. anaemia, could be used to value another seemingly unrelated health state, *provided that the symptoms of this state aligned with the health state description*.

Table A1: Disability weight allocations for TB disease states within GBD 2016 study

		**Drug-susceptible TB**	**Multidrug-resistant TB**	**Extensively drug-resistant TB**	**Latent TB infection**
**Without HIV coinfection**		0.333 *(0.224-0.454)*	0.333 *(0.224-0.454)*	0.333 *(0.224-0.454)*	0 - (asymptomatic)
**HIV Coinfection**	No anaemia	0.408 *(0.274-0.549)*	0.408 *(0.274-0.549)*	0.408 *(0.274-0.549)*	
	With anaemia, mild	0.411 *(0.278-0.551)*	0.411 *(0.278-0.551)*	0.411 *(0.278-0.551)*	
	With anaemia, moderate	0.439 *(0.307-0.577)*	0.439 *(0.307-0.577)*	0.439 *(0.307-0.577)*	
	With anaemia, severe	0.495 *(0.353-0.64)*	0.495 *(0.353-0.64)*	0.495 *(0.353-0.64)*	

Brackets represent 95% uncertainty intervals (UIs) derived from 1000 draws from the posterior distribution of each step in the estimation process - see [26].

Before beginning the study, an initial search within the *Sequela*, *health state name*, and *Health state lay description* fields of the GBD disability weight tables was conducted, using several key terms of interest (Table A2). Subsequently, all 235 unique health states were reviewed, with no further states than those in Table A2 identified.

Table A2: Number of health state descriptions and Sequelae for each of the search terms

**Search term:**	Lung	Respiratory	Breath	Chest	Tuberculosis	TB	Cough*	Wheezing
**Found in name of a specified sequela**	12	43	1	0	16	0	1	0
**Found in unique health state name**	0	81	0	1	15	0	0	0
**Found in any sequela lay description**	0	0	142	4	0	2	38	19
**Unique description in which found**	0	0	14	4	0	1	10	5
**States with at least one search term in any of: sequela, state name, or unique description:**						30		

*Note: search terms are strings, e.g. 'cough' would also return coughing, coughs etc

The thirty health state descriptions of interest from this search, along with the specific sequelae to which they refer are shown in Appendix Table A3. These 30 states were reduced down to 16, based upon the severity of the symptoms and the feasibility of matching these to our cohort data (Table 2). Each of the descriptions was matched as closely as possible to the TB-sequalae reported by study participants.

Table A3: The refined list of health states

Number of specified sequelae covered by health state	Health state name	Health state lay description	GBD Disability Weight (uncertainty interval)
**1**	Acute myocardial infarction, days 3-28	Gets short of breath after heavy physical activity, and tires easily, but has no problems when at rest. The person has to take medication every day and has some anxiety.	0.074 (0.049-0.105)
**25**	Anemia, moderate	Feels moderate fatigue, weakness, and shortness of breath after exercise, making daily activities more difficult.	0.052 (0.034-0.076)
**25**	Anemia, severe	Feels very weak, tired and short of breath, and has problems with activities that require physical effort or deep concentration.	0.149 (0.101-0.209)
**1**	Asthma, controlled	Has wheezing and cough once a month, which does not cause difficulty with daily activities.	0.015 (0.007-0.026)
**1**	Asthma, partially controlled	Has wheezing and cough once a week, which causes some difficulty with daily activities.	0.036 (0.022-0.055)
**1**	Asthma, uncontrolled	Has wheezing, cough and shortness of breath more than twice a week, which causes difficulty with daily activities and sometimes wakes the person at night.	0.133 (0.086-0.192)
**11**	COPD and other chronic respiratory problems, mild	Has cough and shortness of breath after heavy physical activity, but is able to walk long distances and climb stairs.	0.019 (0.011-0.033)
**8**	COPD and other chronic respiratory problems, moderate	Has cough, wheezing and shortness of breath, even after light physical activity. The person feels tired and can walk only short distances or climb only a few stairs.	0.225 (0.153-0.310)
**8**	COPD and other chronic respiratory problems, severe	Has cough, wheezing and shortness of breath all the time. The person has great difficulty walking even short distances or climbing any stairs, feels tired when at rest, and is anxious.	0.408 (0.273-0.556)
**17**	Heart failure, mild	Is short of breath and easily tires with moderate physical activity, such as walking uphill or more than a quarter-mile on level ground. The person feels comfortable at rest or during activities requiring less effort.	0.041 (0.026-0.062)
**17**	Heart failure, moderate	Is short of breath and easily tires with minimal physical activity, such as walking only a short distance. The person feels comfortable at rest but avoids moderate activity.	0.072 (0.047-0.103)
**17**	Heart failure, severe	Is short of breath and feels tired when at rest. The person avoids any physical activity, for fear of worsening the breathing problems.	0.179 (0.122-0.251)

Appendix B: Mapping study SGRQ data to GBD states

Each of the shortlisted health states were disaggregated into the distinct clauses they contained, mindful of words such as 'and' and 'or'. Once split out, each clause was mapped to the respiratory symptoms of each patient using responses of the SGRQ.

Table B1: Mapping patients to health states from GBD using SGRQ responses

**State name**	Health state lay description split into necessary conditions	Mapping procedure
**Acute myocardial infarction, days 3-28**	Gets short of breath after heavy physical activity,	Answered 'Yes' to [playing sports or games makes me feel breathless **OR** My breathing makes it difficult to do things such as very heavy manual work, run, cycle, swim fast or play competitive sports **OR** I get breathless when walking up mountains*]
	and tires easily,	answered 'Yes' to I get exhausted easily
	but has no problems when at rest.	Answered 'No' to feeling breathless when sitting or lying still
	The person has to take medication every day	*Medication criteria not included – see methods*
	and has some anxiety.	Derived variable 'some_anxiety' = 1 *(see bottom of table for more details)*
**Anemia, moderate**	Feels moderate fatigue, weakness, and shortness of breath after exercise,	Answered 'Yes' to I get exhausted easily **AND** I feel breathless playing sports or games **AND** My breathing makes it difficult to do things such as carry heavy loads, dig the garden or shovel snow, jog or walk at 5 miles per hour, play tennis or swim
	making daily activities more difficult.	The derived variable 'inteference_with_activities' = 1
**Anemia, severe**	Feels very weak, tired and short of breath,	Answered 'Yes' to I get exhausted easily
	and has problems with activities that require physical effort	Answered 'Yes' to [I take a long time to get washed or dressed **OR** I cannot take a bath or shower or I take a long time **OR**<br>I walk slower than other people, or I stop for rests **OR**<br>Jobs such as housework take a long time, or I have to stop for rests **OR**<br>If I walk up one flight of stairs, I have to go slowly or stop **OR**<br>If I hurry or walk fast, I have to stop or slow down **OR**<br>My breathing makes it difficult to do things such as walk up hills, carrying things<br>up stairs, light gardening such as weeding, dance, play bowls or play golf **OR**<br>My breathing makes it difficult to do things such as carry heavy loads, dig the<br>garden or shovel snow, jog or walk at 5 miles per hour, play tennis or swim **OR**<br>My breathing makes it difficult to do things such as very heavy manual work,<br>run, cycle, swim fast or play competitive sports.]
	or deep concentration.	*Unable to code (however is OR statement so if above satisfied irrelevant)*
**Asthma, controlled**	Has wheezing and cough once a month,	(Coughing most days a week **OR** several days a week **OR** a few days a month) AND (Wheezing most days a week **OR** several days a week **OR** a few days a month)
	which does not cause difficulty with daily activities.	*(not coded as taking maximum disability weight – see below)*
**Asthma, partially controlled**	Has wheezing and cough once a week,	(Coughing most days a week **OR** several days a week) AND (Wheezing most days a week **OR** several days a week)
	which causes some difficulty with daily activities.	The derived variable 'Inteference_with_activities' = 1

**Asthma, uncontrolled**	Has wheezing, cough and shortness of breath more than twice a week,	(Shortness of breath most days a week **OR** several days a week) **AND** (Coughing most days a week **OR** several days a week) **AND** (Shortness of breath most days a week **OR** several days a week)
	which causes difficulty with daily activities	The derived variable 'Inteference_with_activities' = 1
	and sometimes wakes the person at night.	Answered "My cough and breathing disturbs my sleep"
**COPD and other chronic respiratory problems, mild**	Has cough and shortness of breath after heavy physical activity,	Answered 'Yes' to [‘Playing sports or games makes me feel breathless’ **OR** ‘My breathing makes it difficult to do things such as very heavy manual work, run, cycle, swim fast or play competitive sports’ **OR** ‘I get breathless when walking up mountains’*]
	but is able to walk long distances and climb stairs.	Answered ‘No’ to walking outside on the level makes me breathless
**COPD and other chronic respiratory problems, moderate**	Has cough, wheezing and shortness of breath, even after light physical activity.	Answered ‘Yes’ to ‘My breathing makes it difficult to do things such as walk up hills, carrying things up stairs, light gardening such as weeding, dance, play bowls or play golf’
	The person feels tired and can walk only short distances or climb only a few stairs.	Answered ‘Yes’ to I get exhausted easily **OR** ‘If I walk up a flight of stairs, I have to go slowly or stop’ **OR ‘**Walking outside on the level makes me breathless’ **OR** ‘I walk slower than other people, or have to stop for rests’
**COPD and other chronic respiratory problems, severe**	Has cough, wheezing and shortness of breath all the time.	Over past 3 months has coughed most days a week **OR** had shortness of breath most days a week **OR** had wheezing most days a week
	The person has great difficulty walking even short distances	Answered ‘Yes’ to **‘**Walking outside on the level makes me breathless’ **OR** ‘I walk slower than other people, or have to stop for rests’
	or climbing any stairs,	**OR** (Answers ‘Yes’ to If I walk up one flight of stairs, I have to go slowly or stop **OR** My breathing makes it difficult to do things such as walk up hills, carrying things up stairs, light gardening such as weeding, dance, play bowls or play golf)
	feels tired when at rest,	Answered ‘Yes’ to ‘I get exhausted easily’
	and is anxious.	Derived variable ‘some_anxiety’ = 1 *(see bottom of table for more details)*
**Heart failure, mild**	Is short of breath and easily tires with moderate physical activity, such as walking uphill or more than a quarter-mile on level ground.	Answered 'Yes' to "My breathing makes it difficult to do things such as walk up hills, carrying things up stairs, light gardening such as weeding, dance, play bowls or play golf" **OR** If I walk up one flight of stairs I have to go slowly or stop **OR** ‘I get breathless when walking up mountains’
	The person feels comfortable at rest or during activities requiring less effort.	**AND** answers ‘No’ to **all three** of the following:<br>I feel breathless when sitting or lying still<br>I feel breathless when getting washed or dressed<br>I feel breathless when walking around the home
**Heart failure, moderate**	Is short of breath and easily tires with minimal physical activity, such as walking only a short distance.	Answered 'Yes' to the following questions which relate to their breathing [I walk slower than other people, or I stop for rests **OR** Jobs such as housework take a long time, or I have to stop for rests **OR** If I walk up one flight of stairs, I have to go slowly or stop **OR** If I hurry or walk fast, I have to stop or slow down **OR** I cannot take a bath or shower, or I take a long time **OR ‘**Walking outside on the level makes me breathless’]
	The person feels comfortable at rest	answers ‘No’ to ‘I feel breathless when sitting or lying still’
	but avoids moderate activity.	Answers yes to any of the following:<br>‘I cannot play sports or games’ **OR**<br>‘I cannot go out for entertainment or recreation’ **OR**<br>‘I cannot go out of the house to do the shopping’
**Heart failure, severe**	Is short of breath and feels tired when at rest.	Answers that they feel breathless these days when sitting or lying still **AND** yes to I get exhausted easily
	The person avoids any physical activity, for fear of worsening the breathing problems.	Answers 'Yes' to the following two statements<br>My breathing makes it difficult to do things such as walk up hills, carrying things up stairs, light gardening such as weeding, dance, play bowls or play golf<br>**AND any one of:**

		I cannot do housework **OR**<br>I cannot move far from my bed or chair **OR**<br>I cannot go out of the house to do the shopping
**Derived variable**<br><br>*Interference with Activities*	Derived binary variable used in $State_{ANEMIA\_MOD}$, $State_{ASTHMA\_PAR\_CON}$, and $State_{ASTHMA\_UNCON}$ for conditions relating to difficulties with daily activities<br>*'…had difficulty with daily activities…', '…making daily activities more difficult…', '…which causes some difficulty with daily activities…'.*	*'inteference_with_activities'* = 1 if any two of the following questions were answered 'Yes':<br><br>My cough makes me tired, I am breathless when I talk, my cough or breathing disturbs my sleep, I get exhausted easily, My cough or breathing is embarrassing in public, My chest trouble is a nuisance to my family, friends or neighbours, I have become frail or an invalid because of my chest, Exercise is not safe for me, Everything seems too much of an effort, I take a long time to get washed or dressed, I cannot take a bath or shower, or I take a long time, Jobs such as housework take a long time, or I have to stop for rests, I cannot go out for entertainment or recreation, I cannot go out of the house to do the shopping, I cannot move far from my bed or chair, (My chest trouble made me stop work altogether OR my chest trouble interferes with my work OR made me change my work),<br><br>OR any of My chest condition is the most important problem I have, causes me quite a lot of problems, causes me a few problems, It stops me doing one or two things I would like to do, It stops me doing most of the things I would like to do, it stops me doing everything I would like to do.
**Derived variable**<br><br>*Some Anxiety*	Derived binary variable used in $State_{ACUTE\_MI}$ and $State_{COPD\_SEV}$, for conditions relating to anxiety/worry:<br>'…and has some anxiety…'<br>'…and is anxious…'	*'some_anxiety'* = 1 if any of the following questions were answered 'Yes':<br>'I get afraid or panic when I cannot get my breath' **OR** 'I do not expect my chest to get better' **OR** 'I feel that I am not in control of my chest problem' **OR** 'Exercise is not safe for me'.<br><br>Note: For Baseline, 6-months and 12-months we could have used patients who answered EQ-5D-3L for anxiety/depression as level 2 or 3, however since not available across the full study, the decision was made to only use SGRQ data. See Appendix Tables F6 and F7 for validation of derived variables against EQ-5D responses for T_0-T_{12}.

***Note**: SGRQ Questions about cough, breathlessness, chest trouble etc. ask about what 'usually' makes the respondent breathless/cough/ 'these days', while activities questions ask about how these activities might be affected by breathing.*

Mapping GBD descriptions

Additional considerations of the mapping are detailed below:

Climbing Stairs

While adapting and translating the SGRQ questionnaire for Malawi, the statement '*If I walk up one flight of stairs I have to go slowly or stop'* was deemed not applicable to the population. In the Chichewa version, this was adapted to 'If I walk up a mountain, I have to go slowly or stop', and was accounted for in our mappings.

Minimum Fulfilment Criteria

The aim of this mapping was to calculate DALY weights which represent the *minimum* amount of disability in the population as according to the GBD weights, and accordingly we took the maximum of any satisfied weights. It was therefore unnecessary to map the requirement 'which does not cause difficulty with daily activities', reasoning that regardless of this condition, the disability weight associated would be reached if the other criteria are met. That said, well-defined statements such as 'has no problems when at rest' were mapped (answered 'No' to *'feeling breathless when sitting or lying still'*), to increase the accuracy of our overall classifications.

Applicability of activities

It must be recognised that the activities listed in the SGRQ do not apply to all people, independent of their health (e.g. some people even if healthy would not do housework, and others may not play sports). Therefore, when coding that a patient 'avoids any physical activity' the condition that all of these criteria must be met was relaxed.

Specified Time intervals

Timeframes asked in the SGRQ were closely mapped to those of the GBD descriptions. A conservative approach was taken, with 'a few days a month' not considered to satisfy the requirement of 'once a week'.

Avoidance of activities

In cases that the GBD descriptions state that an individual 'avoids' an activity, we have not been able to map this based on the fact that the activity makes them breathless.

Usual activities

The binary variable '*inteference_with_activities'* was generated based upon responses in the SGRQ questionnaire and used in the mapping of conditions $State_B$, $State_H$, and $State_I$. For T_0, T_6, T_{12}, these derived variables have been validated against the responses to the EQ-5D-3L usual activities question. Appendix Table D7

Anxiety

A binary variable 'some_anxiety' was created, equal to *1* if any of the following questions were answered 'Yes': 'I get afraid or panic when I cannot get my breath', 'I do not expect my chest to get better', 'I feel that I am not in control of my chest problem' or 'Exercise is not safe for me'. For individuals present at T_0, T_6, T_{12}, this was checked against the EQ-5D-3L question on Anxiety/Depression, if this was a level *2* or level *3* (moderate or severe). Appendix Table D6.

Uncertainty intervals

We did not account for the uncertainty intervals of estimates, taking the point-estimate for each state

Appendix C: Proceeding from health states to calculating DALYs

Figure C1: Example of 3-year disability calculated for an individual satisfying four different disability weights, using the 'area under the curve' approach by averaging across timepoints.

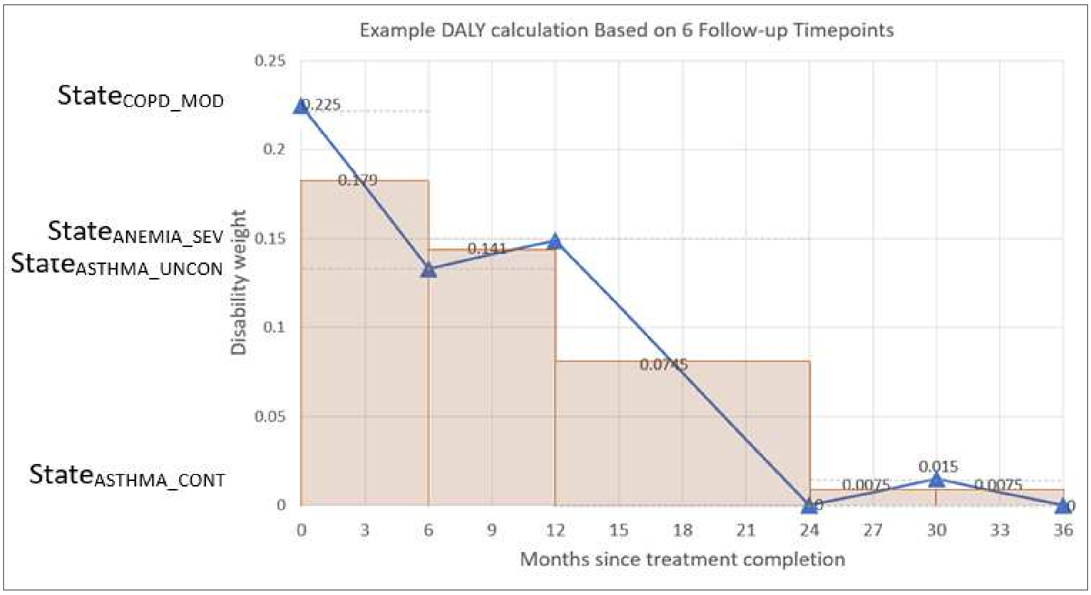

Note that no data were collected at the 18-month timepoint.

Appendix D: Modelling lifetime disability

Several models were fitted to the average (across individuals) SGRQ scores at different timepoints. These included: polynomial models (up to third degree) a log model and a quadratic plateau model. The log model provided an improved fit over polynomial models, both visually and in terms of pseudo R^2, though when used to generate out-of-sample long term predictions, it predicted implausible negative SGRQ values beyond 5 years.

Having observed a plateau in our data towards the end of the three years of data collection, we considered the assumption that HRQoL would stay constant from T_{36} for the remainder of life. This assumption was supported by a quadratic plateau model, which provided the best fit for the data. The model generally fit well and pseudo R^2 was slightly improved compared to the log model. SGRQ was estimated to plateau 3-years post-treatment at an average SGRQ value of 3.066.

Table D1: Wellness of fit of several models fit to SGRQ data across T_0 - T_{36}

Model	Functional form	Pseudo-R^2
Linear model	$y = a + bx$	81.2%
Quadratic model	$y = a + bx + cx^2$	91.6%
Cubic model	$y = a + bx + cx^2 + dx^2$	91.1%
Log model	$y = a + log(x)$	95.2%
Quadratic plateau model	$y = \begin{cases} a + bx - \frac{b}{2x_{crit}} * x^2 & if\ x < x_{crit} \\ a + bx_{crit} - \frac{b}{2x_{crit}} * {x_{crit}}^2 & if\ x \geq x_{crit} \end{cases}$	95.5%

Where $\boldsymbol{x_{crit}}$ denotes a critical $\boldsymbol{x}$ value.

Table D2: Quadratic plateau model estimation

$$\boldsymbol{y = \begin{cases} a + bx - \frac{b}{2x_{crit}} * x^2 & if\ x < x_{crit} \\ a + bx_{crit} - \frac{b}{2x_{crit}} * {x_{crit}}^2 & if\ x \geq x_{crit} \end{cases}}$$

Parameters	Estimate	Standard error	t-value	Pr(>|t|)
$\boldsymbol{a}$	17.006	2.239	7.594	0.00474
$\boldsymbol{b}$	-4.646	1.525	-3.047	0.05558
$\boldsymbol{x_{crit}}$	6.001	1.276	4.702	0.01820
Residual standard error:	*1.175 on 3 degrees of freedom*			

Figure D1: Quadratic plateau model overlaid on mean SGRQ scores at six timepoints of study.

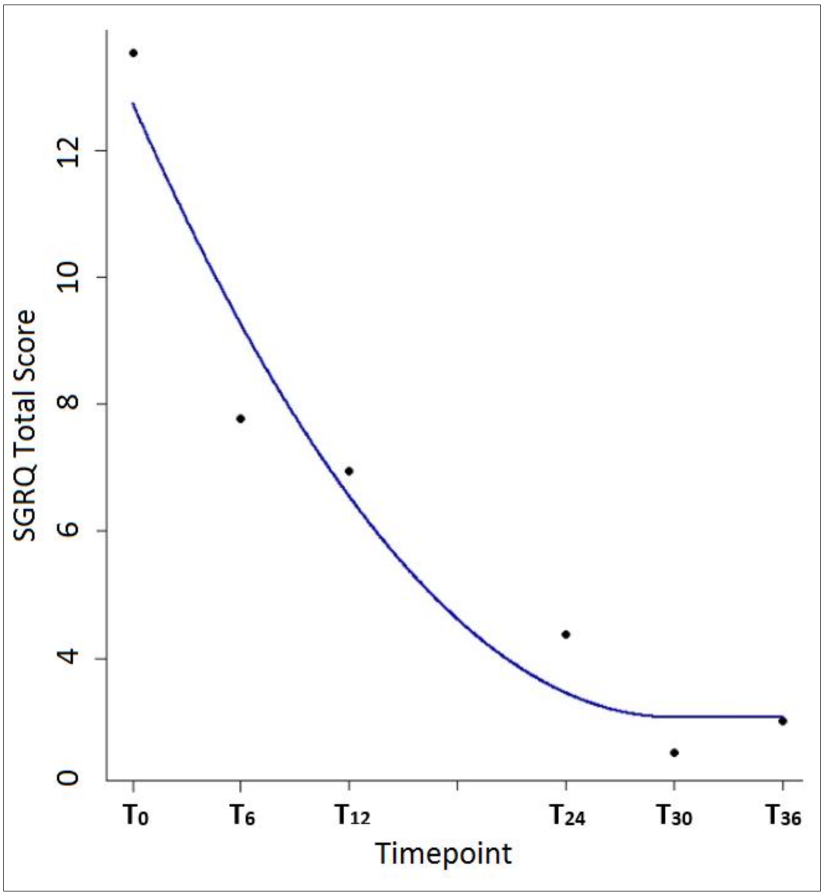

Appendix E: Comparison of active disease and post-TB disability

To frame our findings for post-TB disability, we estimated what disability would likely have been calculated for the population of our core sample during their active tuberculosis disease

Estimates of duration of disease were taken from the technical annex of the WHO 2021 TB report. https://cdn.who.int/media/docs/default-source/hq-tuberculosis/tb-report-2021/technical_annex_methods_2021.pdf (Table 1 page 25). This report uses uniform distributions for duration of treated TB of (0.2−2) for HIV-negative TB patients and (0.01−1) for HIV-positive.
We took the means of these distributions to obtain mean durations of 1.1 years (HIV-) and 0.51 years (HIV+).

The values in column B are GBD disability weights for drug-susceptible TB.

Based upon these values, Columns D and E provide estimates for the mean and total TB-related disability (YLD) of our core sample up to point of treatment. Note, unlike our post-TB estimates, these are not calculated from data collected in this sample but represent estimates similar to those which would be presented in a typical TB cost-utility paper utilising DALYs, wherein primary Health-related Quality of Life data had not been collected. Columns F-through-I are taken from Table 2 in the main manuscript, with Columns J and K showing the percentage increase of disability when our values for the post-TB period are incorporated.

Table E1: Comparison of disability experienced post-TB with that modelled to apply during TB

Column	A*	B†	C	D	E	F	G	H	I	J	K
	Mean duration of treated TB (years)	GBD disability weight of active disease	Number of patients (core sample)	YLD during active disease		YLD 3 years post-TB		Projected post-TB Disability (including 3 year post treatment)		% Increase of disability when including post-TB period	
				Mean	Total	Mean	Total	Mean	Total	3-years only	Lifetime
**Source**	WHO Global TB database	GBD	Our sample	Col**A** * Col**B**	Col**D** * Col**C**	Our study	Our study	Our study	Our study	Col**G** / Col**E**	Col**I** / Col**E**
**HIV-negative:**	1.1	0.333	119	0.37	43.59	0.12	14.68	0.938	111.60	34%	256%
**HIV-positive:**	0.51	0.408	179	0.21	37.25	0.07	13.25	0.445	79.65	36%	214%

*Values in column A are estimates of the duration of TB disease taken from WHO data, which include the period prior to diagnosis and the time on treatment.
†Values in column B are the disability weights allocated to this TB disease period provided by GBD estimates

Appendix F: Additional Tables and Figures

Figure F1: Cohort chart showing deaths known to have occurred between timepoints of study.

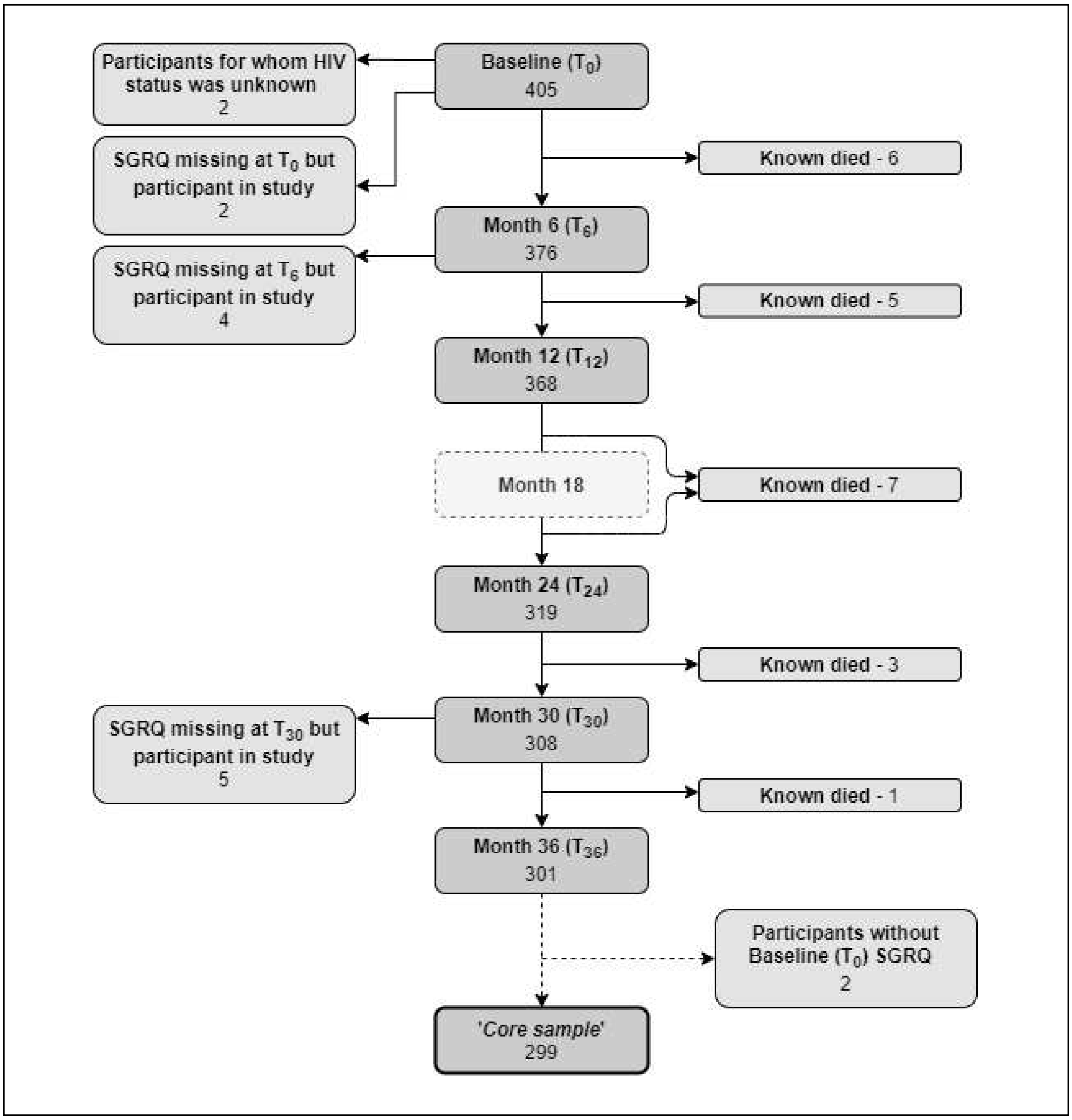

Figure F2: Changes in SGRQ score between time-points, disaggregated by HIV-status and gender

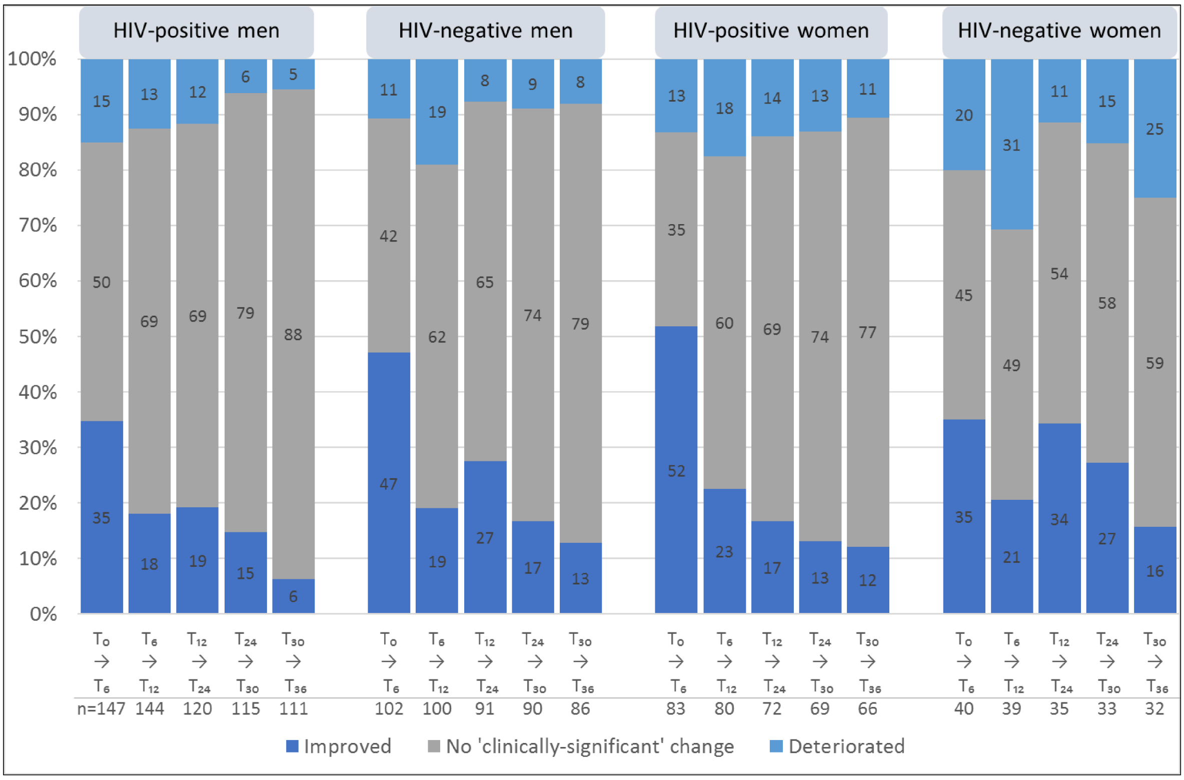

Numbers on bars show percentages. Classification used minimally clinically important difference of 4 SGRQ total score points. Figure demonstrates an increasing number of participants seeing no change in their health over time. The proportion experiencing no change in HRQoL increased between subsequent visits, and in the final period (T_{30}→T_{36}) scores for 80% of participants were unchanged.

Table F1: Changes in St George's Respiratory Questionnaire Total scores between interviews, disaggregated by sex and HIV status

		**T_0 to T_6** (n=372)		**T_6 to T_{12}** (n=363)		**T_{12} to T_{24}** (n=318)		**T_{24} to T_{30}** (n=307)		**T_{30} to T_{36}** (n=295)	
**HIV-positive men**	Improved	51	34.7%	26	18.1%	23	19.2%	17	14.8%	7	6.3%
	**No change**	**74**	**50.3%**	**100**	**69.4%**	**83**	**69.2%**	**91**	**79.1%**	**98**	**88.3%**
	Deteriorated	22	15.0%	18	12.5%	14	11.7%	7	6.1%	6	5.4%
**HIV-negative men**	Improved	48	47.1%	19	19.0%	25	27.5%	15	16.7%	11	12.8%
	**No change**	**43**	**42.2%**	**62**	**62.0%**	**59**	**64.8%**	**67**	**74.4%**	**68**	**79.1%**
	Deteriorated	11	10.8%	19	19.0%	7	7.7	8	8.9%	7	8.1%
**HIV-positive women**	Improved	43	51.8%	18	22.5%	12	16.7%	9	13.0%	8	12.1%
	**No change**	**29**	**34.9%**	**48**	**60.0%**	**50**	**69.4%**	**51**	**73.9%**	**51**	**77.3%**
	Deteriorated	11	13.3%	14	17.5%	10	13.9%	9	13.0%	7	10.6%
**HIV-negative women**	Improved	14	35.0%	8	20.5%	12	34.3%	9	27.3%	5	15.6%
	**No change**	**18**	**45.0%**	**19**	**48.7%**	**19**	**54.3%**	**19**	**57.6%**	**19**	**59.4%**
	Deteriorated	8	20.0%	12	30.8%	4	11.4%	5	15.2%	8	25.0%

Table F2: Allocated health states and maximal disability weights at each time point

Health State ID	Disability Weight	T_0 (n=403)	T_6 (n=376)	T_{12} (n=368)	T_{24} (n=319)	T_{30} (n=308)	T_{36} (n=301)
		Number who met the criteria for a DW at each timepoint (n, %)					
$State_{ASTHMA\_CON}$	**0.015**	20 (5.0%)	12 (3.2%)	8 (2.2%)	9 (2.8%)	4 (1.3%)	5 (1.7%)
$State_{COPD\_MILD}$	**0.019**	211 (52.4%)	101 (26.9%)	86 (23.4%)	50 (15.7%)	38 (12.3%)	44 (14.6%)
$State_{ASTHMA\_PAR\_CON}$	**0.036**	0	2 (0.5%)	0	1 (0.3%)	0	0
$State_{HF\_MILD}$	**0.041**	212 (52.6%)	110 (29.3%)	96 (26.1%)	51 (16.0%)	42 (13.6%)	45 (15.0%)
$State_{ANEMIA\_MOD}$	**0.052**	91 (22.6%)	37 (9.8%)	45 (12.2%)	8 (2.5%)	5 (1.6%)	14 (4.7%)
$State_{HF\_MOD}$	**0.072**	166 (41.2%)	69 (18.4%)	64 (17.4%)	26 (8.2%)	20 (6.5%)	22 (7.3%)
$State_{ACUTE\_MI}$	**0.074**	52 (12.9%)	13 (3.5%)	20 (5.4%)	11 (3.4%)	3 (1.0%)	4 (1.3%)
$State_{ASTHMA\_UNCON}$	**0.133**	0	0	0	0	0	0
$State_{ANEMIA\_SEV}$	**0.149**	112 (27.8%)	43 (11.4%)	49 (13.3%)	13 (4.1%)	5 (1.6%)	10 (3.3%)
$State_{HF\_SEV}$	**0.179**	0	1 (0.3%)	1 (0.3%)	0	0	0
$State_{COPD\_MOD}$	**0.225**	94 (23.3%)	52 (13.8%)	47 (12.8%)	20 (6.3%)	14 (4.5%)	14 (4.7%)
$State_{COPD\_SEV}$	**0.408**	0	0	0	1 (0.3%)	0	0
**Any disability weight**	-	222 (55.1%)	116 (30.9%)	99 (26.9%)	57 (17.9%)	45 (14.6%)	47 (15.6%)

Figure F3: Changes in mean EQ-5D index score and mean VAS score over three-year study period by SGRQ severity quintile at TB treatment completion

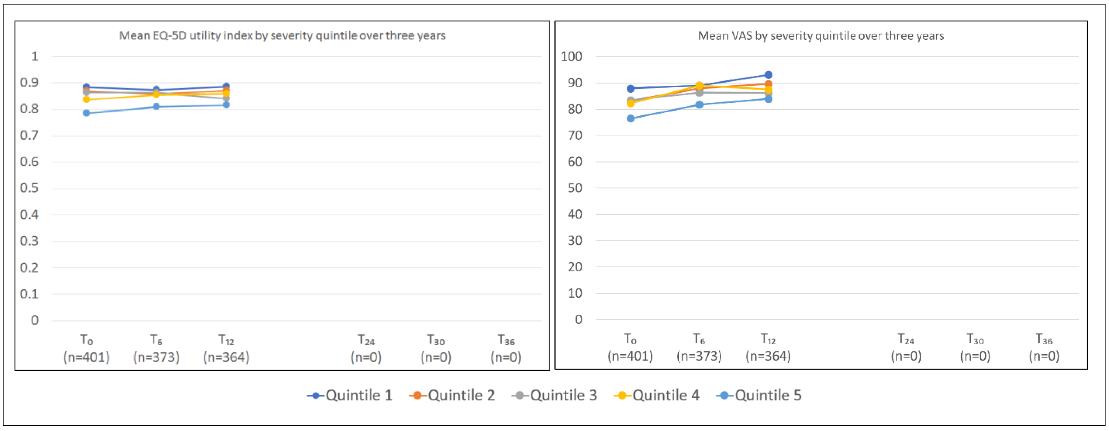

Table F3 : Three-year study period and lifetime projections of YLD within *core sample*, disaggregated by severity within each

		YLD over the three-year follow-up period				Projected Lifetime YLD (including 3 year post treatment)				Disability Weights	
		n	Mean YLD	Total YLD	Burden	n	Mean YLD	Total YLD	Burden	During 3years	After 3years
HIV-positive Men (n=111)	Least severe 20%	41	0.000	0.000	0%	41	0.000	0.000	0%	0.000	0.000
	-	5	0.010	0.051	1%	5	0.010	0.051	0%	0.003	0.000
	-	22	0.035	0.767	10%	22	0.035	0.767	2%	0.012	0.000
	-	22	0.092	2.026	26%	21	0.105	2.215	6%	0.031	0.000
	Most severe 20%	21	0.239	5.024	64%	22	1.579	34.741	92%	0.080	0.045
	**All HIV positive men**	**111**	**0.071**	**7.868**	**100%**	**111**	**0.340**	**37.775**	**100%**	**0.024**	**0.009**
HIV-negative Men (n=86)	Least severe 20%	29	0.000	0.000	0%	29	0.000	0.000	0%	0.000	0.000
	-	6	0.008	0.051	1%	5	0.008	0.040	0%	0.003	0.000
	-	17	0.043	0.727	8%	18	0.047	0.840	2%	0.014	0.000
	-	17	0.136	2.313	24%	17	0.195	3.319	6%	0.045	0.000
	Most severe 20%	17	0.376	6.388	67%	17	2.785	47.349	92%	0.125	0.080
	**All HIV negative Men**	**86**	**0.110**	**9.479**	**100%**	**86**	**0.599**	**51.548**	**100%**	**0.037**	**0.016**
HIV-positive Women (n=68)	Least severe 20%	16	0.000	0.000	0%	16	0.000	0.000	0%	0.000	0.000
	-	15	0.015	0.229	4%	15	0.015	0.229	1%	0.005	0.000
	-	12	0.048	0.572	11%	10	0.047	0.467	1%	0.016	0.000
	-	12	0.095	1.145	21%	14	0.127	1.780	4%	0.032	0.000
	Most severe 20%	13	0.265	3.441	64%	13	3.031	39.397	94%	0.088	0.068
	**All HIV positive women**	**68**	**0.079**	**5.387**	**100%**	**68**	**0.616**	**41.874**	**100%**	**0.026**	**0.013**
HIV-negative Women (n=33)	Least severe 20%	8	0.000	0.000	0%	8	0.000	0.000	0%	0.000	0.000
	-	6	0.021	0.123	2%	6	0.021	0.123	0%	0.007	0.000
	-	6	0.099	0.595	11%	6	0.141	0.846	1%	0.033	0.000
	-	7	0.245	1.718	33%	7	1.535	10.745	18%	0.082	0.060
	Most severe 20%	6	0.461	2.768	53%	6	8.056	48.336	80%	0.154	0.200
	**All HIV negative women**	**33**	**0.158**	**5.204**	**100%**	**33**	**1.820**	**60.049**	**100%**	**0.053**	**0.049**

Mean disability weights for each grouping shaded in grey. Disability Weight quintiles are based on total disability. Two patients who did not provide HIV status are removed from these calculations.

Table F4 – Sensitivity analysis: three-year YLD using Baseline dataset and averages across timepoints

Time period	T_0		T_6		T_{12}		T_{24}		T_{30}		T_{36}
Number of people at timepoint	403		374		366		317		306		299
Sum of individual disability weights at instant	31.646		15.148		14.159		6.609		4.637		4.879
Average individual disability	0.0785		0.0405		0.0387		0.0208		0.0152		0.0163
Averaged disability for interval		0.0595		0.0396		0.0298		0.0180		0.0157	
Length of interval (years)		0.5		0.5		1.0		0.5		0.5	
Length of interval * interval average disability		0.0298		0.0198		0.0298		0.0090		0.0079	
Average 3 year disability (summing row above)					=		0.09619				
**Average 1-year disability (above row / 3)**					**=**		**0.03206**				

Alternative method of disability weight calculation using averages across timepoints. This method includes all patients, who were LTFU during data collection

Figure F4 – Graphical representation of YLD calculation following method which uses all patients.

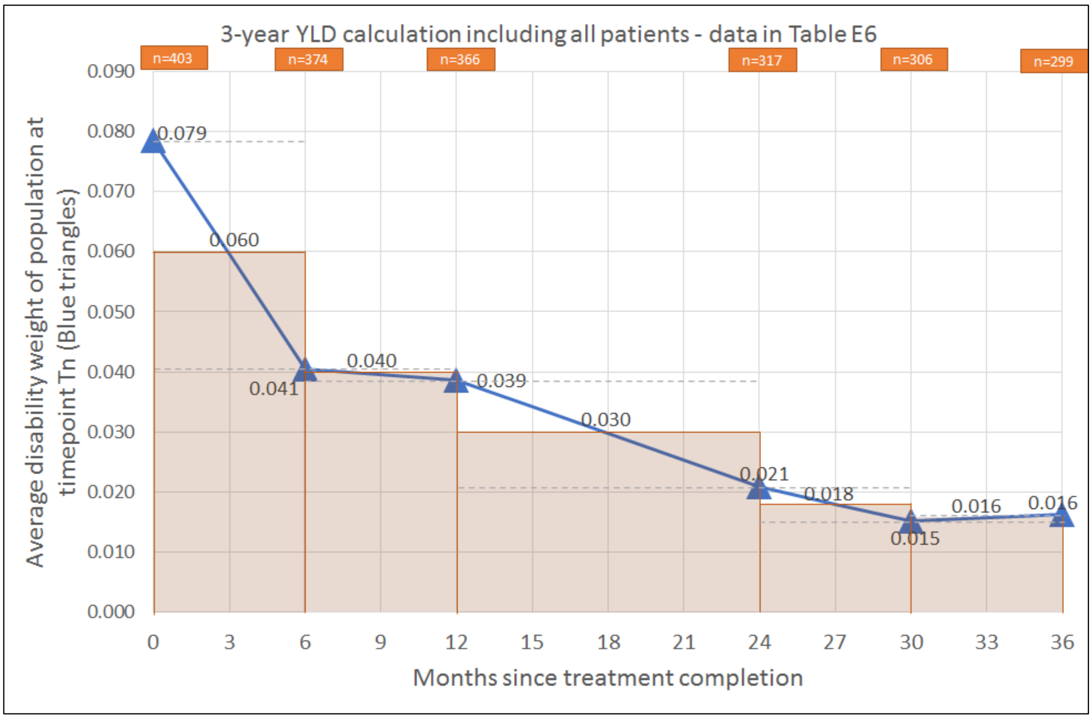

TABLE F5: Sensitivity analysis with less stringent requirements for states across study timepoints - example using controlled, uncontrolled and partially controlled asthma.

State lay description	Coding variation	Frequency	Additional coding	Participants satisfying state T_0	T_6	T_{12}	T_{24}	T_{30}	T_{36}
**Controlled Asthma** (State$_{ASTHMA_CON}$): **DW = 0.015**									
Has wheezing and cough once a month, which does not cause difficulty with daily activities.	Coughing AND Wheeze	most days a week OR several days a week OR a few days a month	-	20	12	8	9	4	5
	Coughing without Wheeze			146	92	61	69	47	45
	Coughing OR Wheeze			159	113	86	73	48	45
**Partially Controlled Asthma** (State$_{ASTHMA_PAR_CON}$): **DW = 0.036**									
Has wheezing and cough once a week, which causes some difficulty with daily activities.	Coughing AND Wheeze	most days a week OR several days a week	AND some difficulty daily activities	0	2	0	1	0	0
	Coughing without Wheeze			11	16	7	7	2	2
	Coughing OR Wheeze			15	16	7	7	2	2
**Uncontrolled Asthma** (State$_{ASTHMA_UNCON}$): **DW = 0.133**									
Has wheezing, cough and shortness of breath [SOB] *more than twice a week, which causes difficulty with daily activities and sometimes wakes the person at night.*	Coughing AND Wheeze AND SOB	most days a week OR several days a week	AND some difficulty daily activities AND cough or breathing disturbs sleep	0	0	0	0	0	0
	(Coughing AND Wheeze) without SOB			0	2	0	0	0	0
	(Coughing OR Wheeze) AND SOB			3	2	1	0	0	0
	(Coughing OR Wheeze) without SOB			11	15	5	0	0	0
	(Coughing AND SOB) without Wheeze			3	2	1	0	0	0
	(Coughing OR SOB) without Wheeze			3	2	1	0	0	0
	(Coughing OR SOB) AND Wheeze			9	17	10	1	2	1
	Cough or Wheeze or SOB			0	2	0	0	0	0

Shaded cells show the stringent coding requirements used in our analyses, clearly leading to conservative estimates. The frequency requirements applied to coding/wheezing/SOB, and 'additional coding' details requirements of all.

Figure F5

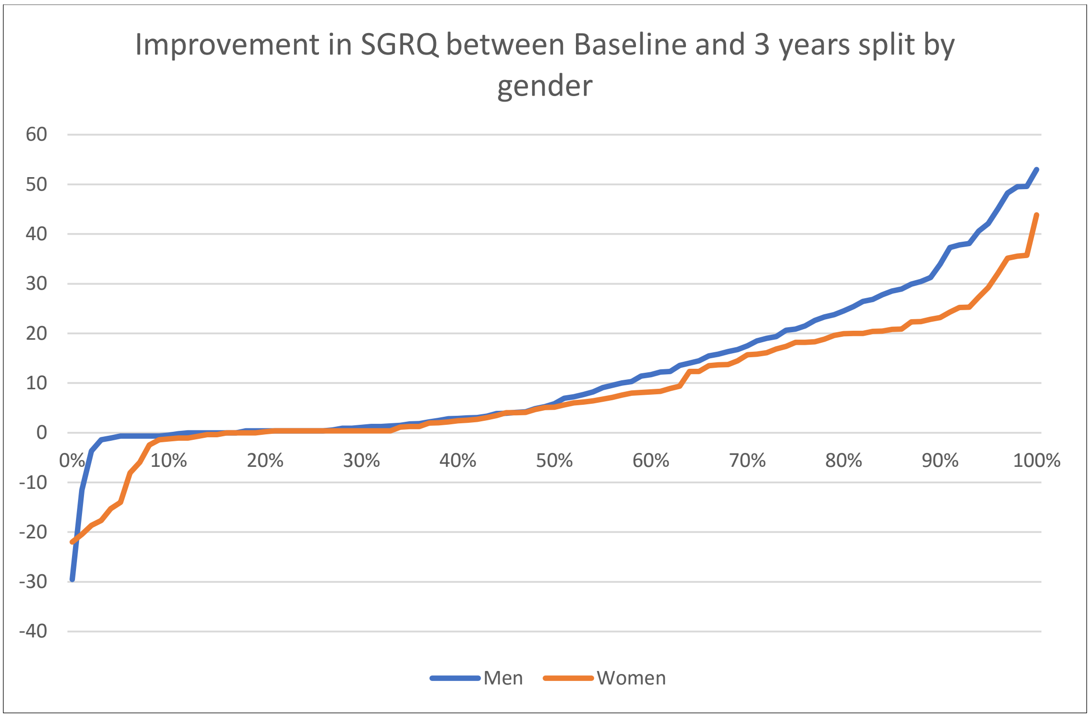

Male and female participants were separately ranked by their improvement in SGRQ total score. While 50% of both men and women improved by 5 or more points, this 50% of highest improving men all saw larger improvements than the 50% of most improved women.

Table F6: Cross checking the derived variable for anxiety against EQ-5D-3L responses in first three timepoints

		EQ-5D-3L anxiety = 1	EQ-5D-3L anxiety = 2 or 3	Total	Sensitivity	Specificity
**Baseline**	some_anxiety == 0	244	100	344	17%	87%
	some_anxiety == 1	38	21	59		
	Total	282	121	403		
**6-month**	some_anxiety == 0	264	94	358	13%	99%
	some_anxiety == 1	3	14	17		
	Total	267	108	375		
**12-month**	some_anxiety == 0	286	59	345	11%	95%
	some_anxiety == 1	14	7	21		
	Total	300	66	366		

some_anxiety = 1 if any of the following questions answered as 'yes':

- I get nervous when I struggle breathing
- I feel that I am not in control of my chest problem
- Physical exercises can't do me any good
- I have no hope that my chest will get better

Note: it would not be expected for this variable to be entirely 'sensitive' when using the EQ-5D-3L responses as this question asks about 'anxiety and depression', whereas our derived variable was aiming to capture 'anxiety' only.

Table F7: Validation of derived variable for Interference with Activities against EQ-5D-3L responses T_0-T_{12}

		EQ-5D-3L usual activities = 1	EQ-5D-3L usual activities = 2 or 3	Total	Sensitivity	Specificity
**Baseline**	Interference with Activities == 0	111	4	115		
	Interference with Activities == 1	228	60	288	94%	33%
	**Total**	**339**	**64**	**403**		
**6-month**	Interference with Activities == 0	211	4	215		
	Interference with Activities == 1	127	33	160	89%	62%
	**Total**	**338**	**37**	**375**		
**12-month**	Interference with Activities == 0	231	5	236		
	Interference with Activities == 1	105	25	130	83%	69%
	**Total**	**336**	**30**	**366**		

'*Interference with Activities*' =1 if at least <u>two</u> of the following are met:

- I get short of breath sitting down or laying down
- I get short of breath getting washed or dressed
- I get short of breath walking around in the house
- I get short of breath walking around outside
- I get short of breath walking up stairs
- I get short of breath walking up hills
- I get short of breath playing sports
- My cough makes me tired
- My cough or breathing is embarrassing in public
- My chest trouble is a nuisance to my family, friends, or neighbours
- My chest condition is the most important problem I have, causes me lots of problems, or causes me a few problems
- My chest stops me doing one or two things I would like to do, most of the things I would like to do, or everything I would like to do
- My chest trouble made me stop work altogether, my chest trouble interferes with my work or made me change my work
